# Identification of Red Grapevine Cultivars (*Vitis vinifera* L.) Preserved in Ancient Vineyards in Axarquia (Andalusia, Spain)

**DOI:** 10.3390/plants9111572

**Published:** 2020-11-13

**Authors:** Ana Jiménez-Cantizano, Alejandro Muñoz-Martín, Antonio Amores-Arrocha, Pau Sancho-Galán, Víctor Palacios

**Affiliations:** Department of Chemical Engineering and Food Technology, Vegetal Production Area, University of Cadiz, Agrifood Campus of International Excellence (ceiA3), IVAGRO, P.O. Box 40, 11510 Puerto Real, Spain; ana.jimenezcantizano@uca.es (A.J.-C.); a.munozmartin@alum.uca.es (A.M.-M.); pau.sancho@uca.es (P.S.-G.); victor.palacios@uca.es (V.P.)

**Keywords:** *Vitis vinifera*, autochthonous grapevine cultivar, cultivar identification, microsatellite marker, ampelographic characterization, somatic variant

## Abstract

A prospecting work at the Axarquia region (Malaga, Spain) was carried out in order to identify local red grapevine cultivars preserved in ancient vineyards. A total of 11 accessions were collected in seven different plots from four municipalities and analyzed using 25 microsatellite loci for cultivar identification. The accessions analyzed were identified as eight different genotypes, seven of them corresponding to known cultivars as ‘Cabernet Sauvignon’, ‘Jaen Tinto’, ‘Molinera’, ‘Monastrell’, ‘Muscat of Alexandria’, ‘Parrel’, and ‘Romé’. In addition, one of them is referred to as the new genotype for ‘Cabriel’ cultivar. Additionally, an ampelographic characterization was carried out with 30 International Organisation of Vine and Wine (OIV) descriptors for two consecutive years for the eight accessions identified as local cultivars. This allowed the identification of a somatic variant of the ‘Muscat of Alexandria’ cultivar that affects the color of the berry and another of ‘Romé’ regarding bunch compactness.

## 1. Introduction

Within the province of Malaga, the Axarquia region is a historically recognized wine territory in Andalusia (Spain). With a mostly steep and mountainous orography, it is located in the most eastern part of the province, spreading along the coast and inland [1]. The cultivation of vines, as well as wine production and trade, have been for a long time the main foundation of the economy of this region of heroic viticulture. Like other Andalusian wine-producing areas, Axarquia has a more thousand-year-old tradition that has not been exempt from the decline that the sector suffered at the end of the last century [2]. The difficult mechanization of the vineyard, the predominance of small vineyard plots, and the low productivity have contributed to vineyard forgetfulness, and nowadays this sector remains rooted in time. Such vineyards may preserve unidentified indigenous or local varieties, which may be of interest in the current viticulture. In this respect, studying their adaptation to warm climatic conditions and their oenological potential to produce new wines could play an important role in the future [3,4]. Besides, nowadays many wine consumers demand new products, with greater diversification and personality; therein lies the growing interest of producers and consumers in ancient local cultivars [5,6].

Since the end of the 19th century, with the phylloxera (*Daktulosphaira vitifoliae*) arrival to Europe, genetic diversity decreased in most European vineyards [7]. In Spain, the first phylloxera outbreak was detected in Malaga (Andalusia) in 1876 [8]. This plague destroyed a large part of the vineyards in this province, which went from 112.872 ha of vineyards in 1878 to 24.180 ha in 1909 [9]. This event gave up a loss of cultivars and consequently of genetic diversity. In historical texts about the region’s viticulture, red grapevine varieties were mentioned such as ‘Cabriel’, ‘Jaén Prieto’, ‘Tempranas Negras’, ‘Alicante’ or ‘Tinto’, ‘Ubíes’, ’Corazón de Cabrito’, ‘Casiles Negras’, ‘Tinto Jaen’, ‘Teta de Negra’, or ‘Cruazno’ [10,11,12]. Actually, a large part of the Axarquia and Malaga vineyard is planted with ‘Muscat of Alexandria’ cultivar for raisin production [13]. Nevertheless, Jiménez-Cantizano et al. [14] in 2014 identified three ancient red cultivars using microsatellite markers: ‘Listán Prieto’, ‘Rome Tinto’, and ‘Jaén Tinto’ collected in vineyards in the province of Malaga.

Nuclear microsatellite markers or simple sequence repeat (SSR) have been widely used to identify and genotype grapevine cultivars [15,16,17,18,19,20,21,22]. In addition, the combination of genetic (microsatellite markers) and ampelographic methods allows the correct identification of cultivars [23]. For this purpose, in old varieties, it is a necessary activity in order to be able to preserve them as plant genetic resources in germplasm banks. Although many projects for the collection and identification of endangered cultivars have been carried out [24,25,26,27,28], there are still old vineyards, located in important wine regions, that have not been prospected. In this way, there are few works that have been developed and published regarding Andalusian ancient cultivars.

The main objective of the present study is the identification of red grapevine cultivars grown in ancient vineyards in the region of the Axarquia (Malaga, Spain). In the scope of this study, the detection of possible synonymies, denomination mistakes, and new genotypes, could contribute to an efficient preservation of old local germplasm that represents valuable genetic combinations for a new viticulture. To this end, a prospection of different ancient local red grapevine cultivars, their genetic analysis using SSR molecular markers, and their morphological description was carried out.

## 2. Results

As a result of the accessions genetic characterization, the presence of a new genotype, a new synonym, and three denomination mistakes were obtained. In order to confirm the identified cultivars based on the molecular results obtained, ampelographic observations were made in the vineyard over two years. In this sense, the ampelographic characterization allowed the identification of two somatic variations for ‘Muscat of Alexandria’ and ‘Romé’ cultivars.

### 2.1. Microsatellite Analysis

Eleven accessions were analyzed at 25 nuclear microsatellite loci resulting in eight nonredundant genotypes (Table 1).

On one hand, M3 and M5 accessions showed the same genotype and, on the other hand, M7, M8, and M10 (Table 2). The nonredundant genotypes obtained were compared with the *Vitis* International Variety Catalogue (*V*IVC) (www.vivc.de) [29] genotype database, Jiménez-Cantizano et al. [30] and Lacombe et al. [31] in order to detect the presence of synonymies, homonymies, and denomination mistakes. The genotypes obtained for the reference cultivars (Supplementary Table S2) were used for testing the microsatellite profiles obtained with the different databases published and comparing the relative allele sizes for the different microsatellite loci. After the comparison with the different databases, seven genotypes were identified with its prime name according to *V*IVC database (Table 2). Genotype III (M3 and M5 samples) has not been identified because it has not been published in the consulted databases. This genotype could be considered a new genotype, and would also correspond to the genotype of the ‘Cabriel’ cultivar identified for the first time. Additionally, the cultivar name was checked in the ampelographic section of the *V*IVC.

Three denomination mistakes were detected for samples M4, M6, and M9, known locally as ‘Romé’, but identified as ‘Monastrell’, ‘Cabernet Sauvignon’, and ‘Parrel’, respectively (Table 2). Furthermore, ‘Casiles Negra’ accession presented a similar genotype of ‘Molinera’ and also, the name ‘Casiles Negra’, is not included in the *V*IVC database. Therefore, ‘Casiles Negra’ should be considered a new synonym of ‘Molinera’ cultivar.

### 2.2. Ampelographic Characterization

**Figure 1:**
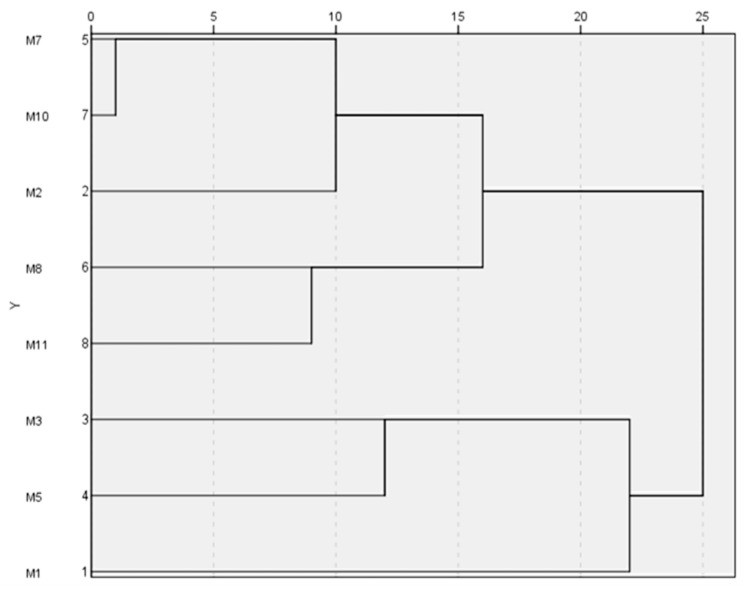
Dendrogram representing the differences among the different studied accessions based on hierarchical cluster analysis (HCA) of ampelographic characterization employing an average link between groups and re-scaled distance cluster combination.

Table 3 shows the results of the morphological characterization of the identified accessions considered as minor Andalusian cultivars. Each accession presented a different phenotype for the 30 evaluated descriptors OIV, except for the accessions M7 and M10 that showed the same phenotype (Table 4, Figure 1) and genotype (Table 1 and Table 2). Nevertheless, M8 accession presented identical genotype at 25 microsatellite loci with M7 and M10 but different phenotype (Table 1). Both accessions are clearly different in the expression of six OIV descriptors (OIV 204, OIV 206, OIV 208, OIV 209, OIV 222, and OIV 238). M7 and M10 have loose bunch and M8 showed very dense bunch. These phenotypic differences detected are those that could allow the establishment of somatic variants or clones in the same cultivar.

‘Moscatel de Alejandría Tinta’ (M2) accession showed the same microsatellite profile with ‘Muscat of Alexandria’, but different berry color; thus, it could be concluded that ‘Moscatel de Alejandría Tinta’ is a red somatic variant for berry color of ‘Muscat of Alexandria’.

## 3. Discussion

During the last 30 years, in Europe, the interest of grapevine growers and wine producers for old and autochthonous cultivars has increased and, therefore, it has become necessary to correctly identify the different cultivars [24]. There are still diverse grapevine synonymies (the same cultivar known under different names) and homonymies (different cultivars known under the same name) to clarify, that alongside with the existence of unnamed accessions, are a source of misidentification and confusion regarding grapevine cultivars designations [27,32,33]. Of the eight genotypes identified in this research work (Table 2), only five correspond to minor Andalusian cultivars (‘Molinera’, ‘Muscat of Alexandria’, ‘Romé’, ’Cabriel’, ‘Jaén Tinto’). These cultivars were cultivated in the province of Malaga at the beginning of the XIX century according Clemente y Rubio [11]. This work has allowed to identify the genotype of the ‘Cabriel’ cultivar for the first time. This genotype is not included in *V*IVC database which aims to virtually assemble all accessions maintained in the existing collections worldwide [34]. In addition, this cultivar is only conserved in Axarquia’s vineyards; accordingly, *V*IVC (www.vivc.de) is not preserved in the different holding institutions.

Additionally, ‘Casiles Negra’ accession presented a similar genotype of ‘Molinera’ cultivar. ‘Casiles’ name is not listed in the *V*IVC database. Nevertheless, García de la Leña [12] cites the ‘Casiles Negras’ cultivar in 1792 among the grapevine cultivars grown in the province of Malaga. Clemente y Rubio [11], in 1807, cited ‘Casiles de Málaga’ cultivar. This result suggests that ‘Casiles Negras’ should be considered as a new synonym of ‘Molinera’.

As for the ampelographic description, this is the methodology that enables the identification of variants or clones in a cultivar [35]. This work has allowed the identification of several somatic variants of local cultivars as they are considered ‘Romé’ and ’Muscat of Alexandria’. In the case of ‘Romé’, the differences found between the accessions studied mainly affect bunch compactness. Grapevine bunch compactness is an economically important trait since it affects several major components of fruit quality. Foremost, compact clusters are more susceptible to pests and diseases [36]. Another somatic variant was detected for ‘Muscat de Alexandria’ cultivar, it is known with the local name ‘Moscatel de Alejandría Tinta’ because it presents red berries. Traditionally, when clones or somatic variants of the same variety have the same phenotypes different enough to be grown for the production of different wines, they are grouped in different cultivars [37] that could keep the name of the progenitor variety [38]. This somatic variant for the berry color of ‘Muscat of Alexandría’ was previously identified by De Lorenzis et al. [39]. They characterized ‘Zibbibo’ (synonymy of ‘Muscat of Alesandría’) and ‘Zibbibo Nero’ and determined that the color locus structure of ‘Zibibbo’ and its putative parents suggested that ‘Zibibbo Nero’ is a berry color revertant of ‘Zibibbo’. In this case, ‘Moscatel de Alejandría Tinta’ and ‘Zibibbo Nero’ would be different names for the same clone. However, the fact that ‘Moscatel de Alejandria Tinta’ and ‘Zibibbo Nero’ have black berries does not mean that they are the same clone but that they can be two different clones with black berries. Another somatic variant for the berry shape has also been described in Andalusia for a ‘Muscat of Alexandria’ accession collected in an ancient vineyard [40].

All these autochthonous cultivars and somatic variants located in the Axarquia region should be studied in order to generate knowledge to make new type of wines. Additionally, it could help to develop strategies to adapt viticulture in different regions to diverse models and markets that nowadays require to ensure the sustainability of the crop. According to Sancho-Galán et al. [41], in order to promote the cultivation of old and autochthonous cultivars, it would be necessary to apply for their inclusion in the Official Register of Authorized Varieties.

## 4. Materials and Methods

### 4.1. Plant Material

A set of 11 grapevine accessions located in seven vineyards of the Axarquia (Malaga, Spain) were studied. All studied accessions were collected and labelled with local names, except the sample accession M3 named as unknown (Supplementary Table S1, Supplementary Figure S1). Six of the accessions (M4, M6, M7, M8, M9, and M10) were named with the same local name, but were located in different vineyard plots. These accessions were analyzed with microsatellite markers and morphologic descriptors. Supplementary Table S1 and Supplemental Figure S1 show the code, location, and the local name accession. Furthermore, six reference grapevine cultivars (‘Airén’, ‘Cabernet Sauvignon’, ‘Chardonnay’, ‘Garnacha’, ‘Pinot noir’, and ‘Syrah’) were also included to test for microsatellite profiles obtained with the different database published [29,30,31].

The morphological descriptions were performed for the eight accessions (M1, M2, M3, M5, M7, M8, M10, and M11) identified as minor Andalusian cultivars.

### 4.2. DNA Extraction and Microsatellite Analysis

Two independent samples were analyzed for each accession. DNA was extracted from wood material using the DNeasy Plant Mini Kit (Qiagen, Hilden, Germany) according to the manufacturer’s instructions. A total of 25 nuclear microsatellite loci were employed to perform the varietal identification following the methodology proposed by Urrestarazu et al. (2015) [42]. PCR mix was carried out in GeneAMP 9700 (Applied Biosystems, Foster City, CA, USA), and the amplified products were separated by capillary electrophoresis, using an automated sequencer ABI PRISM 3130 (Applied Biosystems, Foster City, CA, USA). Fluorescent labelled fragments (6-FAM, VIC, PET, and NED) were detected and sized using GeneMapper v. 3.7, and fragment lengths were assessed with the help of internal standards GeneScan-500 LIZTM (Applied Biosystems, Foster City, CA, USA). The comparison of the SSR obtained was performed using a microsatellite toolkit v. 9.0 software [43]. Lastly, the microsatellite genotypes obtained after the analysis were compared to the genetic profiles given by Jiménez-Cantizano et al. [30] and Lacombe et al. [31], and to the data contained in European grapevine database of microsatellite profiles VIVC [21].

### 4.3. Ampelographic Characterization

A total of 30 OIV descriptors were studied, 17 for mature leaves, six for bunches, and seven for berries (Supplementary Table S3). The morphological characterization was carried out during two consecutive years (2018 and 2019) in field and using a set of 30 descriptors selected from the International Organization of Vine and Wine’s descriptor list [44], including both qualitative and quantitative characteristics, observed or measured in 10 leaves, bunches, and berries. The ampelographic characterization was performed by three different ampelographers, over two years and the modal value is expressed following Benito et al. [45] criteria.

A hierarchical clustering analysis (HCA) using Ward method and the Euclidean square distance was performed, using the statistical software SPSS 24.0 (SPSS Inc., Chicago, IL, USA) to classify clusters according to samples similarity and dissimilarity.

## 5. Conclusions

The plant material that was localized and identified for the first time in this work is a source of interest for the wine sector. Molecular microsatellite analysis allowed the correct identification of the different red grapevine accessions located in ancient vineyards in the Axarquia region. A total eight cultivars were identified in this work of which only five correspond to Andalusian minor local cultivars. In addition, a new genotype was identified for ‘Cabriel’ cultivar. Ampelographic description of the minor local cultivars has contributed to detecting two somatic variants or clones, one for ‘Muscat of Alexandria’ and another one for ‘Romé’.

## Figures and Tables

**Table 1:** Microsatellite profile of 11 grapevine accessions located in Axarquia (Malaga, Spain) analyzed at 25 microsatellite loci.

OIV Code	Accession Code										
	M1	M2	M3	M4	M5	M6	M7	M8	M9	M10	M11
**ssrVrZAG29**	110 110	110 110	110 110	110 110	110 110	110 110	110 110	110 110	110 110	110 110	110 110
**ssrVrZAG62**	187 203	185 203	203 203	187 203	203 203	187 193	187 195	187 195	187 203	187 203	187 195
**ssrVrZAG112**	228 228	233 245	233 236	228 233	233 236	228 233	228 236	228 236	228 236	228 236	231 236
**ssrVrZAG67**	130 150	124 124	137 158	137 137	137 158	124 137	130 158	130 158	137 137	130 158	124 130
**VVMD27**	178 191	176 191	182 191	176 186	182 191	173 186	178 191	178 191	176 186	178 191	178 186
**VVMD5**	231 235	224 228	231 237	222 237	231 237	228 237	235 237	235 237	222 231	235 237	231 237
**VVS2**	135 143	131 148	133 156	131 150	133 156	137 150	135 143	135 143	131 150	135 143	131 143
**ssrVrZAG83**	190 194	188 188	190 190	190 200	190 190	200 200	190 194	190 194	194 200	190 194	190 190
**VVMD28**	233 257	243 266	247 259	243 257	247 259	233 235	235 257	235 257	227 257	235 257	243 247
**VVIh54**	167 169	167 167	167 167	167 167	167 167	167 181	167 167	167 167	167 167	167 167	167 169
**VVIn73**	264 264	264 264	256 264	264 264	256 264	264 268	264 264	264 264	264 264	264 264	264 264
**VMC1b11**	185 188	167 185	188 188	173 188	188 188	185 185	185 188	185 188	173 188	185 188	185 188
**VVMD25**	239 253	247 247	253 254	239 261	253 254	237 247	239 253	239 253	239 261	239 253	239 239
**VVIp31**	186 190	188 190	190 190	180 190	190 190	190 190	176 190	176 190	180 196	176 190	180 192
**VVMD7**	241 247	247 249	231 237	247 247	231 237	237 237	237 237	237 237	237 247	237 237	237 241
**VVIb01**	290 290	290 294	290 306	290 290	290 306	290 290	290 294	290 294	290 290	290 294	290 290
**VVIq52**	84 88	82 82	84 88	88 88	84 88	82 88	82 88	82 88	84 88	82 88	84 88
**VVMD24**	210 210	212 212	208 208	208 217	208 208	208 217	208 208	208 208	208 208	208 208	208 208
**VVIp60**	317 321	317 321	321 321	317 321	321 321	305 313	317 325	317 325	317 325	317 325	317 321
**VVMD32**	250 270	262 270	248 250	238 254	248 250	238 238	254 270	254 270	238 248	254 270	254 256
**VVIn16**	150 152	148 150	150 150	152 158	150 150	152 152	152 152	152 152	152 158	152 152	150 152
**VMC4f3.1**	166 186	180 206	182 206	178 178	182 206	172 178	186 188	186 188	178 178	186 188	172 186
**ssrVrZAG79**	244 254	244 252	244 254	248 258	244 254	244 244	244 254	244 254	240 258	244 254	240 244
**VVMD21**	248 248	255 265	242 255	242 248	242 255	248 257	242 248	242 248	248 255	242 248	248 248
**VVIv67**	371 375	375 389	357 375	357 365	357 375	365 371	365 375	365 375	365 365	365 375	361 365

**Table 2:** Genotypes identified for the 11 grapevine accessions characterized at 25 microsatellite loci.

Genotype	Code Accession	Local Name	Prime Name *
I	M1	Casiles Negra	MOLINERA
II	M2	Moscatel de Alejandría Tinta	MUSCAT OF ALEXANDRIA
III	M3, M5	Unknown/Cabriel	-
IV	M4	Romé	MONASTRELL
V	M6	Romé	CABERNET SAUVIGNON
VI	M7, M8, M10	Romé	ROMÉ
VII	M9	Romé	PARREL
VIII	M11	Jaén Tinto	JAEN TINTO

* Prime name according to *V*IVC (www.vivc.de).

**Table 3:** Ampelographic characteristics of 30 OIV descriptors on grapevine accessions located in Axarquia (Malaga, Spain).

Accession Code								
OIV Code	M1	M2	M3	M5	M7	M8	M10	M11
OIV 065	9	5	7	5	5	5	5	5
OIV 067	3	3	3	3	3	3	3	3
OIV 068	3	3	3	2	3	3	3	3
OIV 070	1	2	1	2	1	1	1	1
OIV 071	1	2	1	2	1	1	1	1
OIV 072	1	5	5	1	3	3	3	1
OIV 074	2	2	2	2	2	2	2	1
OIV 076	5	2	5	5	3	3	3	2
OIV 079	3	7	3	3	7	7	7	3
OIV 080	2	3	3	3	2	2	2	1
OIV 081-1	1	1	1	1	1	1	1	1
OIV 081-2	1	1	1	1	1	1	1	1
OIV 082	4	1	1	1	3	3	3	1
OIV 083-1	2	3	3	3	2	2	2	3
OIV 083-2	1	1	1	1	1	1	1	1
OIV 084	1	3	1	1	7	7	7	7
OIV 085	1	3	1	1	3	3	3	3
OIV 202	7	5	7	5	5	5	5	7
OIV 203	7	3	5	3	5	5	5	7
OIV 204	3	3	1	5	3	9	3	9
OIV 206	5	3	7	5	5	3	5	5
OIV 208	2	2	1	1	2	1	2	2
OIV 209	3	3	3	2	2	1	2	2
OIV 220	9	7	5	5	5	5	5	5
OIV 221	5	5	3	3	5	5	5	5
OIV 222	7	1	1	2	1	2	1	2
OIV 223	3	3	3	3	2	2	2	2
OIV 225	5	3	5	5	5	5	5	5
OIV 238	5	5	5	7	7	5	7	5
OIV 241	3	3	3	3	3	3	3	3

**Table 4:** Number of different observations between different accessions characterized with 30 OIV descriptors.

	M1	M2	M3	M5	M7	M8	M10
**M2**	16						
**M3**	12	14					
**M5**	16	14	12				
**M7**	14	14	17	17			
**M8**	16	15	16	18	6		
**M10**	14	14	17	17	0	6	
**M11**	13	15	15	15	12	9	12

## Supplementary Materials

The following are available online at https://www.mdpi.com/2223-7747/9/11/1572/s1, Figure S1. Vineyard map location. Table S1. Code and local name accession, location, and municipality of the vineyard in Malaga (Spain). Table S2. Microsatellite profile of six reference cultivars analyzed at 25 microsatellite loci. Table S3. Code, description, and scale of the 30 OIV descriptors selected for the ampelographic characterization.
